# Omental adipocytes promote peritoneal metastasis of gastric cancer through the CXCL2–VEGFA axis

**DOI:** 10.1038/s41416-020-0898-3

**Published:** 2020-05-22

**Authors:** Makoto Natsume, Takaya Shimura, Hiroyasu Iwasaki, Yusuke Okuda, Kazuki Hayashi, Satoru Takahashi, Hiromi Kataoka

**Affiliations:** 1 Department of Gastroenterology and Metabolism, Nagoya City University Graduate School of Medical Sciences, 1 Kawasumi, Mizuho-cho, Mizuho-ku Nagoya, 467-8601 Japan; 2 Department of Experimental Pathology and Tumor Biology, Nagoya City University Graduate School of Medical Sciences, 1-Kawasumi, Mizuho-cho, Mizuho-ku Nagoya, 467-8601 Japan

**Keywords:** Gastric cancer, Cancer microenvironment

## Abstract

**Background:**

Gastric cancer (GC) patients frequently develop peritoneal metastasis; however, the underlying mechanism remains unknown. We hypothesised that omental adipocytes (OmAd) trigger GC cells towards malignant activity to induce peritoneal metastasis.

**Methods:**

We analysed interactions among human GC cells, endothelial cells and OmAd using a 3D co-culture system. We also employed a multipronged animal study, including subcutaneous and orthotopic tumours, and humanised omental adipose tissue models. Urinary levels of CXCL2 were analysed in human GC patients with and without peritoneal metastasis.

**Results:**

Conditioned media derived from OmAd (OmAd-CM) promoted the proliferation, migration and capacity to induce angiogenesis of GC cells through AKT phosphorylation and VEGFA overexpression, whereas silencing CXCL2 in OmAd cancelled OmAd-induced effects. In an orthotopic tumour model using SCID mice, omentectomy suppressed GC growth and peritoneal dissemination, and reduced serum levels of CXCL2. OmAd promoted GC growth in a humanised omental adipose tissue model using NSG mice, but silencing CXCL2 in OmAd cancelled OmAd-induced tumour growth. Finally, urinary levels of CXCL2 were significantly higher in GC patients with peritoneal metastasis than in those without.

**Conclusion:**

Omental adipocytes trigger GC cells to an aggressive phenotype through CXCL2 secretion, which induces angiogenesis followed by cell growth and peritoneal metastasis.

## Background

Gastric cancer (GC) is the sixth most common malignancy and second leading cause of cancer deaths worldwide,^1^ indicating that its control is a global challenge. While GC diagnostic and treatment techniques have developed in recent years, the 5-year survival rate of stage IV GC remains at <15% with a poor prognosis.^2^ Peritoneal metastasis is a common metastatic pattern of GC,^3^ which represents not only a poor prognostic factor for GC but also reduces patients’ quality of life due to the induction of pain, intestinal obstruction and ascites. However, despite the recent development of diagnostic imaging including contrast-enhanced computed tomography and ^18^F-fluoro-2-deoxyglucose positron emission tomography, diagnostic sensitivity is very low for peritoneal metastasis.^4^ In this context, the diagnosis and regulation of peritoneal metastasis are the most critical issues for GC. However, since the mechanism by which GC frequently causes peritoneal metastasis remains unknown, no specific therapies have yet been established.^5,6^

A variety of stromal cells are contained in the microenvironment around cancer cells, and they play critical roles in the enhancement of cancer growth and metastatic dissemination.^7^ Adipocytes in fat tissues also regulate cancer development by their effects on the microenvironment. It is well known that obesity is involved in the incidence and prognosis of various malignancies in light of strong evidence from prospective studies.^8–10^ Since adipocytes are major components of adipose tissue, many studies have reported on the relationship between adipocytes and cancer cell growth to elucidate the mechanism of obesity-inducible cancer development^11^ in tissues, such as the breast,^12,13^ prostate,^14^ and pancreas.^15^ However, the exact mechanism of how obesity increases the cancer risk remains a subject of study. In addition, whether adipocytes play differential roles depending on localisation remains to be elucidated.

The greater omentum is the largest of the peritoneal tissues, and hangs down from the greater curvature of the stomach and covers most of the abdominal organs. It is generally considered that the main roles of the greater omentum are to protect the abdominal organs from outside impacts and prevent the intra-abdominal spread of inflammation. Importantly, the greater omentum is the most favourable site where GC exhibits peritoneal dissemination. On the other hand, angiogenesis is the most critical event for cancer development,^16^ as well as being involved in obesity.^17^ Angiogenesis is thus a common inducible factor for both cancer and adipose tissue development.

It is noteworthy that the greater omentum consists of abundant adipose tissue and occupies a large part of the visceral fat. In addition, the greater omentum is highly vascularised. Moreover, the recent studies reported that omental adipocytes promote cell proliferation and peritoneal metastasis in ovarian cancer.^18,19^ Consequently, we speculated that omental adipocytes might play crucial roles in the development of GC peritoneal metastasis via angiogenesis and secreted growth factors.

We thus conducted this study to clarify the relationship between omental adipose tissue and peritoneal dissemination of GC. We herein provide novel evidence that omental adipocytes facilitate the growth and peritoneal dissemination of GC through the activation of angiogenesis.

## Methods

### Cell culture

First, we screened for GC cell lines derived from primary tumours, not metastatic sites, using databases, as GC cells from metastatic sites might have already undergone some alterations. We identified two cell lines, AGS (ATCC, Manassas, VA) and IM95 (JCRB cell bank, Osaka, Japan), as non-metastatic human GC cells for use in this study. AGS was maintained in Ham’s F-12 with L-glutamine and phenol red medium (WAKO, Osaka, Japan) with 10% foetal bovine serum (FBS). IM95 was maintained in D-MEM (high glucose) with L-glutamine and phenol red (WAKO) with 10% FBS and 1% insulin (Thermo Fisher Scientific, Waltham, MA).

Human microvascular endothelial cells (HMVECs) (Lonza, Basel, Switzerland) and human omental precursor adipocytes (OmPrAd) (Zen-Bio, Inc., Research Triangle, NC) were also used in this study. HMVECs were cultured in EGM-2 medium (Lonza, Basel, Switzerland) on plates coated with an attachment factor protein (Thermo Fisher Scientific). In some conditions, HMVECs were cultured in EBM-2 medium (Lonza) that was a basal medium without any growth factors. Human OmPrAd were cultured in high glucose D-MEM with 10% FBS and 20 ng/ml fibroblast growth factor-basic (R&D Systems, Minneapolis, MN). OmPrAd were differentiated to mature omental adipocytes (OmAd) with FBS 10% high glucose D-MEM containing 1 μM dexamethasone (Sigma-Aldrich, St. Louis, MO), 1 μM insulin, 2 μM rosiglitazone (Sigma-Aldrich) and 0.5 mM 3-isobutyl-1-methylxanthine (Sigma-Aldrich). OmAd cells were maintained in 10% FBS high glucose D-MEM supplemented with 1 μM insulin.

### Cell proliferation assays

Cells were seeded at 5.0 × 10^3^ cells/well on 96-well plates and cultured overnight. Next, cells were washed with PBS and cultured in the 1% FBS medium of each condition: conditioned media of control and OmAd (OmAd-CM). Cell proliferation was measured using a Cell Counting Kit-8 assay (Dojindo Laboratories, Kumamoto, Japan).

### Migration assay

A migration assay was performed using Corning™ transwell plates with 8.0-μm pore membranes (top chamber) for the 24-well culture plate (Corning Inc., Corning, NY). GC cells were seeded at a density of 50,000 cells with 0% FBS D-MEM in the top chamber of the 24-well plate. Media in the bottom chamber was either 0% FBS control media or individual OmAd-CMs. After incubation for 24 h (AGS) or 48 h (IM95), migrating cells were fixed with 10% formalin, stained with crystal violet and counted with microscopy.

### Endothelial cell recruitment assay

An endothelial cell (EC) recruitment assay was also performed using the same transwell plates as a migration assay. GC cells from each condition were seeded at a density of 100,000 cells on the bottom wells of a 24-well plate. Following overnight incubation, cells were washed with PBS and cultured in serum starved media for 24 h. Media from the bottom chamber were replaced with 0% FBS control media or individual OmAd-CMs. After 24 h, HMVECs (50,000 cells/well) in 0.1% FBS EBM-2 were placed in the top chamber coated with an attachment factor, and the media in the bottom chamber were also replaced with the same media, 0.1% FBS EBM-2. After incubation for 24 h, HMVECs that had migrated toward GC cells through the top chamber membrane were fixed with 10% formalin and counted with microscopy after staining.

### Tube-formation assay

AGS and IM95 cells were seeded at 5.0 × 10^5^ cells/well on a six-well plate, and after 6 h, the cells were washed with PBS and cultured in serum-free media overnight. Next, the culture media were changed to FBS 0% control media or individual CMs. After 24-h stimulation of GC cells, cells were washed with PBS and cultured in FBS 0% EBM-2 media. Finally, individual CMs from GC cells were collected after 24 h. Each CM was used for the tube-formation assay after preparing 1% FBS CM.

Incubated HMVECs were washed with PBS and added to serum-free medium. After 2 h of serum starvation, HMVECs were added to 1% FBS CM from the individual GC cell conditions, and 15,000 cells/well were placed in 96-wells coated with Matrigel growth factor reduced membrane matrix, phenol red-free, LDEV-free (Corning Inc.). The chambers were incubated with each CM for 6 h, photographed under a microscope and branching numbers were counted. Cells with more than three branches were defined as having tube formation.

### Arrays

The TaqMan human angiogenesis array (Thermo Fisher Scientific), which covers 92 angiogenic genes, is a qPCR based comprehensive analysis, which was used to discover critical angiogenic factors in GC cells. Human Cytokine Antibody Array C1000 (RayBiotech, Inc., Norcross, GA), covering 120 proteins, was employed to identify aberrant secreted proteins between control media and OmAd-CMs according to the manufacturer’s instructions.

### RNA extraction and qPCR

Each RNA was extracted according to the manufacturer’s instructions. The total mRNA from GC cells was extracted using a simplyRNA Tissue Kit (Promega Corporation, Madison, WI). In addition, mRNA extraction of tumour tissues removed from mice was performed using a RNeasy Mini (QIAGEN, Venlo, Netherlands). cDNA was synthesised using a High Capacity cDNA Reverse Transcription Kit (Thermo Fisher Scientific) and a PCR Thermal Cycle Dice (Takara, Shiga, Japan). RT-PCR was performed using power up SYBR green Master mix (Thermo Fisher Scientific). Cycling conditions were as follows: one cycle each at 50 °C for 2 min and 95 °C for 2 min, and 45 cycles each at 95 °C for 15 sec and 60 °C for 1 min. RT-PCR was performed using a 7500 Fast Real-Time PCR system (Applied Biosystems, Foster City, CA) according to the manufacturer’s recommendations. Data were normalised to β-actin. The breakdown of the specific primers is shown in Supplementary Table 1.

### RNA interference knockdown

siRNA transfection was performed using a Lipofectamine RNAiMAX (Thermo Fisher Scientific) according to the manufacturer’s instructions. OmAd cells were transfected with the desired siRNA using siGENOME non-targeting siRNA (siNT) control pool and siGENOME human C–X–C motif chemokine ligand 2 (CXCL2) siRNA SMART pool (siCXCL2) (Dharmacon, Lafayette, CO). FBS 0% medium was used to culture each OmAd for 24 h, and each conditioned media (CM) was collected, name as OmAd-CM, siNT OmAd-CM and siCXCL2 OmAd-CM, respectively.

### Transfection

To establish GC cells with stable luciferase expression, transfection to AGS cells was performed with EF1a-Luciferase (firefly)-2A-GFP (puro) (Gen Target, San Diego, CA) according to the manufacturer’s instructions. After transfection, stable successful transfection could be monitored with GFP.

To establish OmAd cells with stable silencing of CXCL2, OmPrAd cells were transfected through short-hairpin RNA (shRNA) delivery with SMART vector non-targeting hEF1a-TurboRFP Control Particles or SMART vector Lentiviral Human CXCL2 hEF1a-Turbo RFP (Dharmacon) using polybrene (2 µg/ml) according to the manufacturer’s instructions. After transfection, stable successful transfection could be monitored with RFP. Each Om(Pr)Ad with shRNA was named as shNT-Om(Pr)Ad and shCXCL2-Om(Pr)Ad, respectively.

These transfected cells were selected and maintained in media containing 0.4 µg/ml puromycin.

### Animal experiments

The protocols for all animal studies were approved by Nagoya City University Center for Experimental Animal Science, and the mice were housed according to the guidelines of Nagoya City University for Animal Experiments.

The mice were acclimatised for 2 weeks before the experimental procedures, and kept in individual cages with free access to water and food. All mice were maintained under specific pathogen-free conditions with a 12-h light/dark cycle. The 6-week-old female SCID mice (Charles River Laboratories Japan, Inc., Yokohama, Japan) were used for the subcutaneous tumour and orthotopic tumour models. Inhalation of CO_2_ was used for all mice euthanasia.

- *Orthotopic tumour model:*
  At the age of 8 weeks, SCID mice underwent laparotomy; the omentum was excised in the omentectomy group, whereas the omentum was left intact in the omentum-preserved group. Next, 1 × 10^7^ AGS cells with Matrigel (Corning Inc.) were orthotopically transplanted in the stomach of each mouse. Mice weights were measured twice a week. Four weeks after transplantation, tumours were excised and fixed in formalin or frozen in liquid nitrogen for immunohistochemical staining and qPCR. The ascites was collected and quantified. Blood samples were collected before the mice were euthanised.
- *Subcutaneous tumour model:*
  At the age of 8 weeks, 1 × 10^7^ AGS cells treated with control media, siNT OmAd-CM or siCXCL2 OmAd-CM were subcutaneously injected into SCID mice  with each media containing Matrigel growth factor reduced membrane matrix. The maximum tumour diameter (L) and the diameter at right angles to that axis (W) were measured twice weekly using callipers, and the volume was calculated according to the formula (L × W^2^)/2. The weight of mice was also measured twice weekly. At the age of 16 weeks, blood samples were collected before the mice were euthanised. After euthanasia, transplanted tumours were excised and fixed in formalin or frozen in liquid nitrogen for immunohistochemical staining and qPCR.
- *Humanised omental adipose tissue model:*
  In order to create a humanised omental adipose tissue model, 6-week-old male NSG mice (Charles River Laboratories Japan Inc.) capable of engraftment at high rates in human normal cells were used. A portion of 3 × 10^5^ RFP-labelled shNT-OmPrAd or shCXCL2-OmPrAd cells was subcutaneously injected into the left flank of NSG mice. One week later, 1 × 10^7^ AGS cells were subcutaneously injected in the vicinity.

Bioluminescence was measured using a multifunctional in vivo imaging system (IVIS; MIIS, Molecular Devices, Tokyo, Japan) to confirm GC cell growth. To visualise the tumours in mice, IVIS was performed as follows. The mice were anesthetised and subsequently subjected to intraperitoneal injection with D-luciferin (150 mg/g body weight, WAKO) in PBS. Two minutes later, a monochrome photograph was acquired, followed immediately by bioluminescence acquisition for 5 min and in vivo fluorescence imaging. Quantification was conducted using MetaMorph-MIIS software (Molecular Devices) to monitor tumour growth. The bioluminescence images were exported as false-coloured images using matched visualisation scales. At the end of the experiment, mice were sacrificed, and transplanted tumours were excised and fixed in formalin or frozen in liquid nitrogen for immunohistochemical staining and qPCR.

### Enzyme-linked immunosorbent assays

We measured protein concentrations of CXCL2, CXCL3 and VEGFA via mono-specific enzyme-linked immunosorbent assays (ELISAs), SimpleStep ELISA kits (Abcam), according to the manufacturer’s instructions. Mouse CXCL2 ELISA kit (ab204517) (Abcam) was used to analyse the serum level of CXCL2 in orthotopic tumour model, whereas Human CXCL2 ELISA kit (ab184862) (Abcam) was used for other analyses of CXCL2. Human CXCL3 (ab234574) and VEGFA (ab222510) ELISA kits (Abcam) were also used to analyse CXCL3 concentration in OmAd-CM and urinary VEGFA concentration in GC patients, respectively.

### Patients and urine samples

All GC patients were histologically diagnosed as GC by biopsy sample. As previously described,^20–23^ all urine samples were collected before treatment of GC, immediately frozen, and stored at −80 °C until assay. Urine samples from 75 patients with non-metastatic GC and 13 patients with peritoneal metastasis were analysed in this study. Patient data were retrieved from computerised databases and reviewed retrospectively.

The study protocol was conducted according to the ethical guidelines of the 1975 Declaration of Helsinki (6th revision, 2008). All patients provided their written, informed consent before study entry.

### Statistical analysis

Data were analysed using the Mann–Whitney *U* test, Student's *t* test or two-factor ANOVA, as appropriate. *P* < 0.05 was considered to be statistically significant. Data analyses were performed in IBM SPSS statistics, version 25 (IBM Corp., Tokyo, Japan).

## Results

### Omental adipocyte-derived conditioned media promote proliferation, migration and the ability to induce angiogenesis of GC cells

**Fig. 1:**
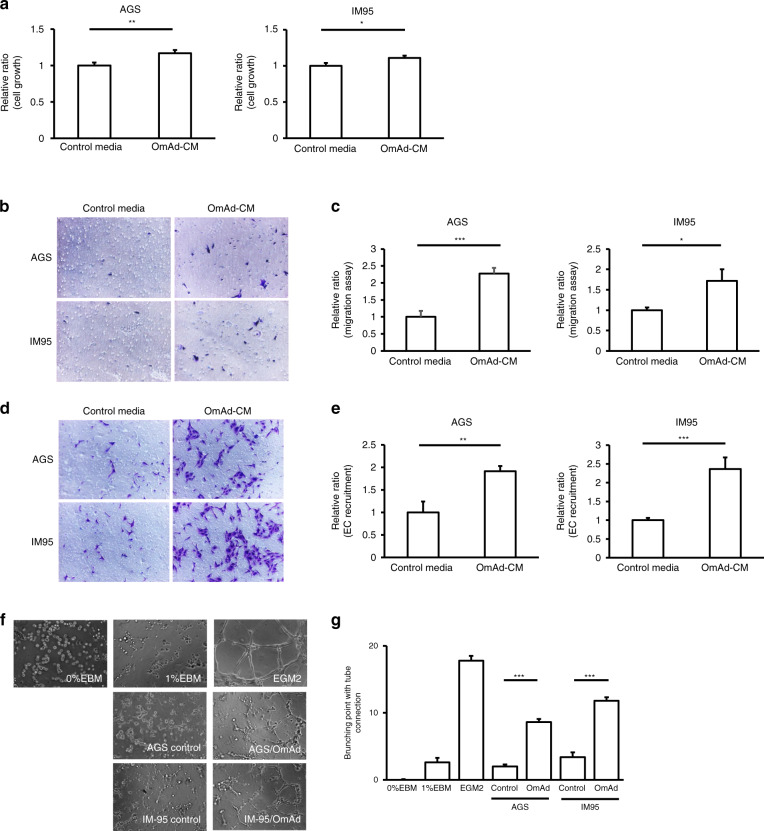
Omental adipocytes induce GC cell growth/migration and in vitro angiogenesis. Each graph represents the mean ± SE from three independent experiments. *P < 0.05; **P < 0.01; ***P < 0.001. **a** Cell growth. AGS and IM95 were used as GC cell lines. The relative ratios are shown of absorbance for OmAd-CM to those for control media (*n* = 5). Mean, AGS: 1.0 (control), 1.2 (OmAd-CM); IM95: 1.0 (control), 1.2 (OmAd-CM). **b** Representative images of migration assay (×100). **c** Quantification of migration assay. Migrated GC cells were counted from averages at four microscopic fields, and each result was presented as the mean of at least three independent experiments. Each value represents the mean relative ratio of migrated GC cells for OmAd-CM to that for control media. Mean, AGS: 1.0 (control), 2.3 (OmAd-CM); IM95: 1.0 (control), 1.7 (OmAd-CM). **d** Representative images of EC recruitment assay (×100). **e** Quantification of EC recruitment assay. Migrated HMVECs were counted from averages at four microscopic fields, and each result was presented as the mean of at least three independent experiments. Each value represents the mean relative ratio of migrated HMVECs co-cultured with OmAd-CM-treated GC cells to those co-cultured with control-treated GC cells. Mean, AGS: 1.0 (control), 1.9 (OmAd-CM); IM95: 1.0 (control), 2.4 (OmAd-CM). **f** Representative images of tube-formation assay (×100). **g** Quantification of tube-formation assay. EBM-2 with FBS 0 and 1% were used as negative controls and EGM2 was used as a positive control. Each value represents the mean number of branched tubes under each condition. Mean, 0 (0% EBM), 2.6 (1%EBM), 17.8 (EGM2); AGS: 2.0 (control), 8.6 (OmAd); IM95: 3.4 (control), 11.8 (OmAd).

First, we investigated the impact of OmAd on non-metastatic primary GC cells by analysing cell growth and migration, which are necessary steps in tumour progression. OmAd-CM significantly increased GC cell growth compared with control media in both GC cell types (Fig. 1a). OmAd-CM significantly promoted migration of both AGS and IM95 cells compared with control media (Fig. 1b, c).

Next, we analysed the impact of OmAd on angiogenesis, another critical step for tumour development and the switch to an active phenotype, using endothelial cell (EC) recruitment and tube-formation assays. Both GC cells treated with OmAd-CM significantly increased EC recruitment, an indispensable step in tumour angiogenesis, compared with control GC cells (Fig. 1d, e). Conditioned media derived from GC cells treated with OmAd-CM showed significantly increased tube formation compared with those from control GC cells (Fig. 1f, g).

These results suggest that OmAd-CM enhances proliferation and migration of GC cells as well as promotes an angiogenic phenotype.

### GRO is critical for omental adipocytes-induced malignant activation of GC cells

In order to identify the causative secreted protein in OmAd-CM, we performed a human cytokine antibody array. It was demonstrated that GRO is more abundant in OmAd-CM than in the control media. Interestingly, no difference was found for GROα levels between OmAd-CM and the control media (Supplementary Fig. 1a).

OmAd-CM increased GC cell growth, but inhibition of GRO in OmAd-CM by a neutralising antibody significantly suppressed the capacity for GC cell growth for both AGS and IM95 GC cells (Supplementary Fig. 1b). Similarly, the anti-GRO antibody significantly suppressed OmAd-CM-induced cell migration in both GC cells (Supplementary Fig. 1c, d).

OmAd-CM increased the ability of GC cells to recruit EC, whereas OmAd-CM with the anti-GRO antibody significantly suppressed the ability of GC cells to recruit ECs compared with OmAd-CM with control IgG (Supplementary Fig. 1e, f).

Furthermore, OmAd-CM containing anti-GRO antibody significantly suppressed the OmAd-CM-induced capacity of GC cells to induce EC tube formation compared with OmAd-CM containing control IgG (Supplementary Fig. 1g, h). Anti-angiogenic effects via GRO inhibition were observed in both AGS and IM95 GC cell lines. These results suggest that GRO levels in the OmAd-CM are critical for malignant activation of GC cells.

### CXCL2 in omental adipocytes transforms GC towards an aggressive phenotype via VEGFA induction in GC cells

From the above experiments, GRO in OmAd-CM promoted GC cell proliferation, migration and angiogenesis. Next, we attempted to identify the most critical factor among the GRO family, which includes CXCL1 (GROα), CXCL2 (GROβ) and CXCL3 (GROγ).

**Fig. 2:**
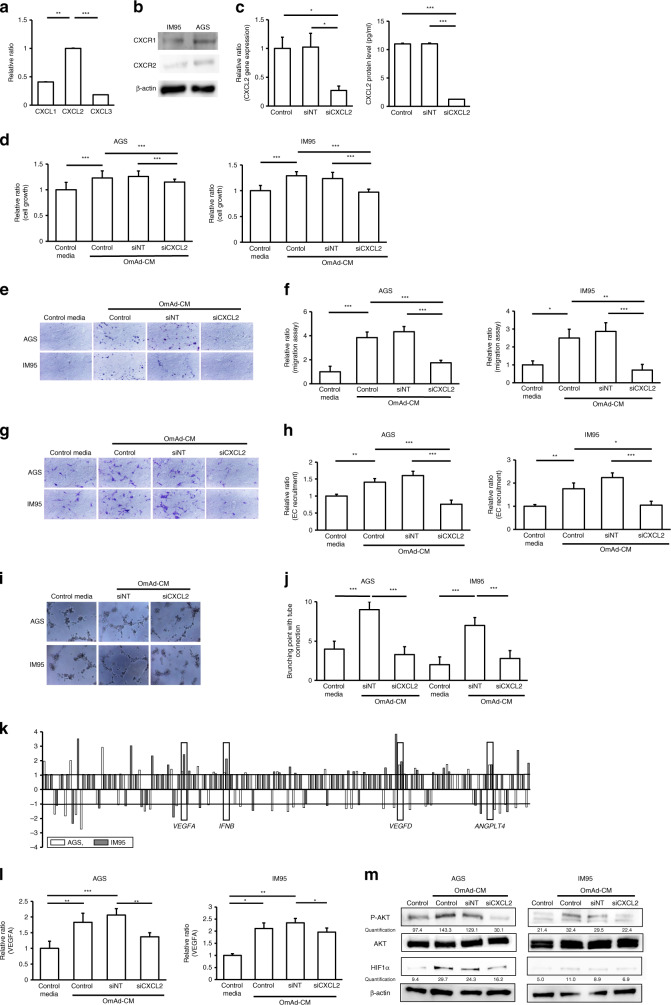
CXCL2 in omental adipocytes is critical for GC cell growth/migration and in vitro angiogenesis. Each graph represents the mean ± SE from three independent experiments. *P < 0.05; **P < 0.01; ***P < 0.001. **a** Gene expression of GRO family in omental adipocyte. Bar graph represents the relative ratio of each RNA expression to CXCL2 expression level, using 2^−ΔΔCt^, where ΔCt indicates the difference in Ct values between each gene and β-actin [ΔCt = Ct (target gene) − Ct (β-actin)]. Mean, 0.41 (CXCL1), 1.0 (CXCL2), 0.18 (CXCL3). **b** Western blotting**. c** Efficiency of siRNA. Omental preadipocytes were transfected with non-targeting siRNA and CXCL2. The left graph represents RNA gene expression of CXCL2 in the OmAd cells. Bar graph represents the relative ratio of siNT and siCXCL2 to control, using 2^−ΔΔCt^, where ΔCt indicates the difference in Ct values between each gene and β-actin [ΔCt = Ct (target gene) − Ct (β-actin)]. The right graph represents protein concentration of CXCL2 in the OmAd-CM. Mean, RNA level: 1.0 (control), 1.0 (siNT), 0.27 (siCXCL2); protein level: 11.0 (control), 11.0 (siNT), 1.3 (siCXCL2). **d** Cell growth. Shown are the relative ratios of absorbance under each condition of OmAd-CM to those under control media (*n* = 5). Mean, AGS: 1.0 (control), 1.2 (control OmAd-CM), 1.3 (siNT OmAd-CM), 1.1 (siCXCL2 OmAd-CM); IM95: 1.0 (control), 1.3 (control OmAd-CM), 1.2 (siNT OmAd-CM), 1.0 (siCXCL2 OmAd-CM). **e** Representative images of migration assay (×100). **f** Quantification of migration assay. Migrated GC cells were counted from averages at four microscopic fields, and each result was presented as the mean of at least three independent experiments. Each value represents the mean relative ratio of migrated GC cells under each type of OmAd-CM to those under control media. Mean, AGS: 1.0 (control), 3.8 (control OmAd-CM), 4.3 (siNT OmAd-CM), 1.7 (siCXCL2 OmAd-CM); IM95: 1.0 (control), 2.5 (control OmAd-CM), 2.9 (siNT OmAd-CM), 0.7 (siCXCL2 OmAd-CM). **g** Representative images of EC recruitment assay (×100). **h** Quantification of EC recruitment assay. Migrated HMVECs were counted from averages at four microscopic fields, and each result was presented as the mean of at least three independent experiments. Each value represents the mean relative ratio of migrated HMVECs co-cultured with GC cells treated with each type of OmAd-CM to those co-cultured with GC cells treated with control media. Mean, AGS: 1.0 (control), 1.4 (control OmAd-CM), 1.6 (siNT OmAd-CM), 0.7 (siCXCL2 OmAd-CM); IM95: 1.0 (control), 1.8 (control OmAd-CM), 2.2 (siNT OmAd-CM), 1.0 (siCXCL2 OmAd-CM). **i** Representative images of tube-formation assay (×100)**. j** Quantification of tube-formation assay. Each value represents the mean number of branched tubes under each condition. Mean, AGS: 4.0 (control), 9.0 (siNT OmAd-CM), 3.3 (siCXCL2 OmAd-CM); IM95: 2.0 (control), 7.0 (siNT OmAd-CM), 2.8 (siCXCL2 OmAd-CM). **k** Semi-comprehensive analysis for angiogenic factors. RNA was extracted from both AGS and IM95 cells before and after OmAd-stimulation. Each bar represents the relative ratios of each gene expression in OmAd-treated GC cells to those in non-treated GC cells. **l** *VEGFA* expression in GC cells. Relative ratios are expressed with 2^−ΔΔCt^, where ΔCt indicates the difference in Ct values between each gene and β-actin. Mean, AGS: 1.0 (control), 1.8 (control OmAd-CM), 2.1 (siNT OmAd-CM), 1.4 (siCXCL2 OmAd-CM); IM95: 1.0 (control), 2.1 (control OmAd-CM), 2.4 (siNT OmAd-CM), 1.9 (siCXCL2 OmAd-CM). **m** AKT phosphorylation and HIF1α expression in GC cells. Each protein extracted from GC cells incubated with control media, OmAd-CM, siNT OmAd-CM or siCXCL2 OmAd-CM for 24 h was immunoblotted with anti-phospho-AKT, anti-AKT and anti-HIFα antibodies. Each band density was quantified with Image J. β-actin is shown as a loading control.

qPCR was conducted on RNA extracted from OmAd, and showed significantly higher CXCL2 expression than CXCL1 and CXCL3 (Fig. 2a). This result was consistent with the data of secreted protein in Supplementary Fig. 1a, which indicated that GROα was not abundant in the OmAd-CM. Moreover, CXCL2 also showed significantly higher protein level compared with CXCL3 that was undetectable range in the OmAd-CM. We confirmed both GC cells expressed the CXCL2 receptors CXCR1 and CXCR2 (Fig. 2b). Next, siRNA was used to silence CXCL2 in OmAd, resulting in a significant reduction in CXCL2 gene and protein expression in the siCXCL2 compared with the control and siNT conditions (Fig. 2c).

OmAd-CM increased GC cell growth; however, siCXCL2 OmAd-CM significantly decreased GC cell proliferation compared with control OmAd-CM and siNT OmAd-CM (Fig. 2d). Similarly, siCXCL2 OmAd-CM significantly suppressed OmAd-CM-induced migration compared with control and siNT OmAd-CM in both GC cell lines (Fig. 2e, f). Likewise, in the in vitro angiogenesis assay, OmAd-CM silencing CXCL2 also significantly suppressed the OmAd-CM-induced capacity of GC cells to induce EC recruitment (Fig. 2g, h) and tube formation (Fig. 2i, j) compared with control and siNT OmAd-CM in both GC cells. These results indicate that siCXCL2 in the OmAd is critical for obtaining the OmAd-induced aggressive potential of GC.

In order to elucidate how CXCL2 from OmAd activates GC cells, we conducted an angiogenesis array by comparing RNA expression of control GC cells with that of OmAd-CM-treated GC cells, using both AGS and IM95 cells. The results of the angiogenesis arrays revealed that the OmAd-CM-treated cells showed higher gene expressions of *VEGFA*, *IFNB*, *VEGFD* and *ANGPTL4* compared with the control cells, and revealed consistent results in both of the GC cell lines (Fig. 2k). We also found that adipocytes deficient in CXCL2 lost the ability of OmAd-CM to induce *VEGFA* overexpression in both GC cells (Fig. 2l). In contrast, no consistent results were observed for *IFNB*, *VEGFD* and *ANGPTL4* gene expression (data not shown).

To identify the mechanism by which CXCL2 from OmAd-CM regulates VEGFA, we analysed the level of HIF1α, a key transcription factor for VEGFA, as well as the AKT pathway. AKT phosphorylation was increased, and HIF1α was overexpressed after OmAd-CM treatment regardless of control and siNT transfection; however, siCXCL2 OmAd-CM suppressed AKT phosphorylation and HIF1α overexpression (Fig. 2m). On the other hand, stimulation by OmAd-CM did not modulate MAPK signalling in GC cells (data not shown). These results suggested that CXCL2 secreted from OmAd induces VEGFA expression through AKT phosphorylation and HIF1α overexpression in GC cells, resulting in promotion of cancer cell growth/migration and angiogenesis.

### Omentum enhances GC growth and peritoneal metastasis in orthotopic tumour model

In vitro analysis demonstrated that OmAd promoted GC cell proliferation/migration and the ability of GC cells to induce EC recruitment and tube formation, suggesting that OmAd contributes to the aggressive transformation of GC as well as the induction of angiogenesis.

First, in order to analyse whether the omentum could promote GC growth, invasion and peritoneal dissemination, an orthotopic in vivo model was employed using omentum-preserved and omentectomised SCID mice.

**Fig. 3:**
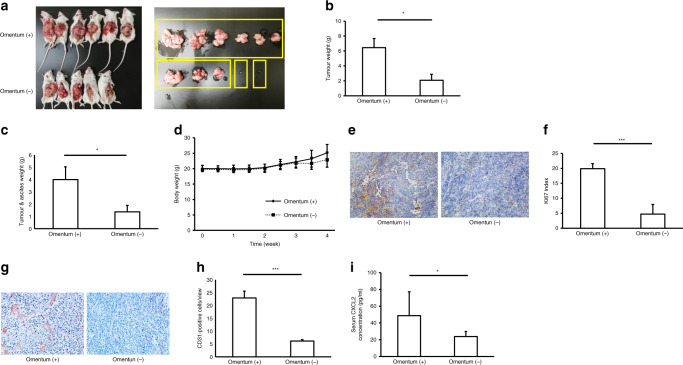
Omentum enhances GC growth and peritoneal metastasis in an orthotopic tumour model. Omentum-preserved group (*n* = 6); omentectomy group (*n* = 5). *P < 0.05; **P < 0.01; ***P < 0.001. **a** Representative macroscopic images of GC tumours in the omentum-preserved and omentectomy groups of SCID mice. SCID mice were subjected to laparotomy: the omentum-preserved group, in which the omentum was intact, and the omentectomy group, in which the omentum was excised, and then 1 × 10^7^ GC cells were injected into the stomach wall. Data are shown as tumours at 4 weeks after implantation. **b** GC growth in the omentum-preserved and omentectomy groups. Each bar represents the average weight of gastric tumours that were removed at 4 weeks after injection. Mean, 6.5 (omentum (+)), 2.1 (omentum (−)). **c** Peritoneal metastatic tumour and ascites. Each bar represents the average weight of peritoneal tumours and ascites collected at 4 weeks after injection. Mean, 4.0 (omentum (+)), 1.4 (omentum (−)). **d** Body weight curves**. e** Representative images of Ki67 immunohistochemistry (×200). **f** Ki67 index. Each bar represents the average rate of Ki67-positive cells in GC tumour tissues (**×**400). Mean, 19.9 (omentum (+)), 4.7 (omentum (−)). **g** Representative images of microvessel density (×200). **h** Tumour angiogenesis. Each bar represents the average number of CD31-positive microvessels in GC tumour tissues (**×**400). Mean, 22.9 (omentum (+)), 6.1 (omentum (−)). **i** Serum CXCL2 levels. Each bar represents the average serum CXCL2 level of mice. Mean, 48.8 (omentum (+)), 23.9 (omentum (−)).

The GC tumour volume and weight were significantly increased in the omentum-preserved group compared with the omentectomy group (Fig. 3a, b). Mice in the omentum-preserved group had significantly more peritoneal metastatic tumour and ascites weight compared with the omentectomy group (Fig. 3c). On the other hand, no significant differences were noted for body weight between the two groups (Fig. 3d). Tumour tissues in the omentum-preserved group showed significantly higher intensity of Ki67 staining compared with the omentectomy group (Fig. 3e, f). In addition, tumours in the omentum-preserved group significantly promoted angiogenesis compared with the omentectomy group (Fig. 3g, h). Moreover, serum levels of CXCL2 were significantly higher in the omentum-preserved group than in the omentectomy group (Fig. 3i).

### CXCL2 in omental adipocytes is critical for GC growth in a xenograft model

Results of the orthotopic GC model suggested that the omentum plays crucial roles in the development of GC growth and peritoneal dissemination. The in vitro study revealed that the CXCL2–VEGFA axis between OmAd and GC cells was critical for OmAd-induced GC malignant transformation.

**Fig. 4:**
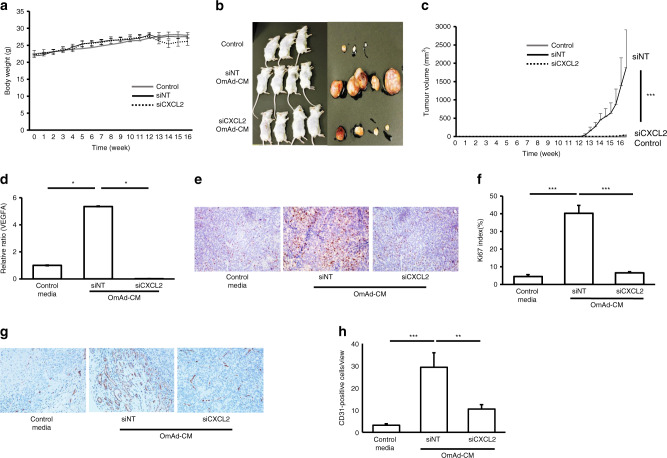
CXCL2 in omental adipocytes is critical for GC growth in a xenograft model. Control group: AGS cells treated with control media were subcutaneously injected with control media (*n* = 13). siNT OmAd-CM group: AGS cells treated with siNT OmAd-CM were subcutaneously injected with siNT OmAd-CM (*n* = 18). siCXCL2 OmAd-CM group: AGS cells treated with siCXCL2 OmAd-CM were subcutaneously injected with siCXCL2 OmAd-CM (*n* = 5). **a** Body weight curves. **b** Representative macroscopic images of resected tumours treated with control media, siNT OmAd-CM and siCXCL2 OmAd-CM in SCID mice. **c** Tumour growth curve. Tumour size and volume were measured twice per week. **d** *VEGFA* expression in tumour tissues. Each graph bar represents the relative ratios of 2^−ΔΔCt^ of siNT OmAd-CM and siCXCL2 OmAd-CM to that of control media. Mean, 1.0 (control), 5.4 (siNT OmAd-CM), 0.1 (siCXCL2 OmAd-CM). **e** Representative images of Ki67 immunohistochemistry (×200). **f** Ki67 index. Each bar represents the average rate of Ki67-positive cells in GC tumour tissues (**×**400). Mean, 4.4 (control), 40.2 (siNT OmAd-CM), 6.5 (siCXCL2 OmAd-CM). **g** Representative images of tumour angiogenesis (×200). **h** Quantification of tumour angiogenesis. Each bar represents the average number of CD31-positive microvessels in GC tumour tissues (**×**400). Mean, 3.3 (control), 29.3 (siNT OmAd-CM), 10.5 (siCXCL2 OmAd-CM).

For further validation, we performed an animal study using the following xenograft model. The AGS cells treated with control media, siNT OmAd-CM and siCXCL2 OmAd-CM were subcutaneously injected with each media into SCID mice. No significant difference was found in the body weight of mice among the three groups (Fig. 4a). Tumour volume was significantly increased in the siNT OmAd-CM-stimulated GC group compared with the control GC group, whereas siCXCL2 silencing of OmAd significantly abolished the OmAd-CM promotion of tumour growth (Fig. 4b, c). Analysis of RNA gene expression of *VEGFA* in tumour tissues at 13 weeks, which is the early phase of tumour development, indicated that *VEGFA* expression was significantly higher in the siNT OmAd-CM-treated GC tumour than in the control GC tumour. On the other hand, siCXCL2 OmAd-CM significantly decreased *VEGFA* expression in the tumour compared to siNT OmAd-CM (Fig. 4d). Similarly, OmAd-CM treatment significantly increased immunoreactivity to Ki67 in the GC tumour tissues, but CXCL2 silencing of OmAd significantly suppressed the ability of OmAd to increase Ki67 staining (Fig. 4e, f). Likewise, OmAd-CM treatment significantly facilitated tumour angiogenesis, but CXCL2 silencing of OmAd significantly suppressed the angiogenic ability of OmAd (Fig. 4g, h). These results also demonstrated that OmAd is critical for GC tumour growth through the CXCL2–VEGFA axis in the xenograft tumour model.

### Omental adipocytes promote GC growth through the CXCL2 axis in a humanised omental adipose tissue model using NSG mice

To corroborate these findings and firmly establish the critical function of CXCL2 in omental adipocyte-induced GC development, we established omental preadipocytes in which CXCL2 expression was stably knocked down using specific shRNAs (shCXCL2-OmPrAd), as well as non-targeting knockdown (shNT-OmPrAd). After injection of each OmPrAd into NSG mice, we established a microenvironment that mimicked the human omentum.

**Fig. 5:**
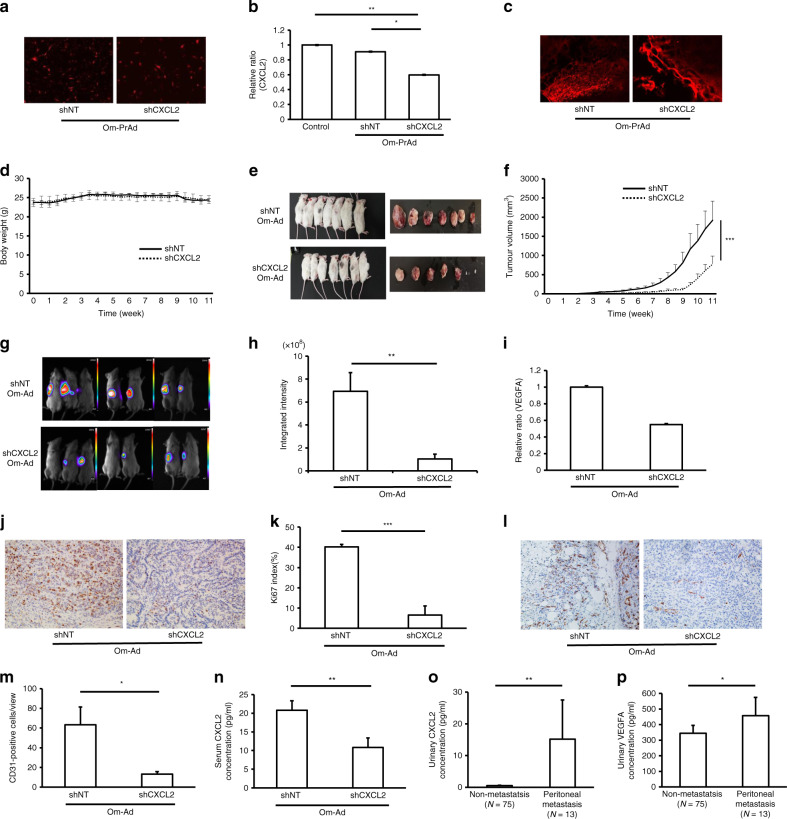
Omental adipocytes facilitate GC growth through the CXCL2–VEGFA axis in a humanised omental adipose tissue model using NSG mice. RFP-labelled shNT-OmPrAd or shCXCL2-OmPrAd cells was subcutaneously injected into the left flank of NSG mice. One week later, AGS cells were subcutaneously injected in the vicinity. shNT-OmAd group (*n* = 7); shCXCL2-OmAd group (*n* = 7). Each graph represents the mean ± SE from three independent experiments. *P < 0.05; **P < 0.01; ***P < 0.001. **a** Red fluorescent protein expression in omental preadipocytes. RFP was detected in OmPrAd transfected with shNT and shCXCL2. **b** Efficiency of shRNA. Omental preadipocytes were transfected with shRNA of non-targeting genes and CXCL2. Bar graph represents the relative ratio of shNT and shCXCL2 to control using 2^−ΔΔCt^ by qPCR. Mean, 1.0 (control), 0.95 (shNT-OmPrAd), 0.59 (shCXCL2-OmPrAd). **c** Humanised omental adipose tissues. RFP was detected in adipose tissues where shNT-OmPrAd or shCXCL2-OmPrAd was subcutaneously injected. **d** Body weight curves. **e** Representative macroscopic images of GC tumours. Excised tumours were shown at 11 weeks after transplantation of GC cells. **f** Tumour growth curve. Tumour size and volume were measured twice per week. **g** Representative bioluminescence images. **h** Quantification of bioluminescence signals. Total luminescence flux from the tumour region was quantified using MetaMorph-MIIS software. Mean, 6.9 × 10^8^ (shNT-OmAd), 1.0 × 10^8^ (shCXCL2-OmAd). **i** *VEGFA* expression in tumour tissues. *VEGFA* expression levels were analysed using qPCR in tumour tissues. Each graph bar represents the relative ratio of 2^−ΔΔCt^ of the shCXCL2-OmAd group to that of shNT-OmAd. Mean, 1.0 (shNT-OmAd), 0.55 (shCXCL2-OmAd). **j** Representative images of Ki67 immunohistochemistry (×200). Representative images of Ki67 immunostaining in tumour tissues. **k** Ki67 index. Each bar represents the average rate of Ki67-positive cells in GC tumour tissues (**×**400). Mean, 40.2 (shNT-OmAd), 6.5 (shCXCL2-OmAd). **l** Representative images of tumour angiogenesis (×200). **m** Quantification of tumour angiogenesis. Each bar represents the average number of CD31-positive microvessels in GC tumour tissues (**×**400). Mean, 63.4 (shNT-OmAd), 13.2 (shCXCL2-OmAd). **n** Serum CXCL2 level in NSG mice. Each bar represents the average serum CXCL2 level in the shNT-OmAd and shCXCL2-OmAd groups. Mean, 20.8 (shNT-OmAd), 10.8 (shCXCL2-OmAd). **o** Urinary level of CXCL2 in human GC patients. Each bar represents the average urinary level of CXCL2 in GC patients with or without peritoneal metastasis. Mean, 0.55 (non-metastasis), 15.2 (peritoneal metastasis). **p** Urinary level of VEGFA in human GC patients. Each bar represents the average urinary level of VEGFA in GC patients with or without peritoneal metastasis. Mean, 344 (non-metastasis), 457 (peritoneal metastasis).

Immunofluorescent microscopy revealed that RFP was properly introduced into both shNT-OmPrAd and shCXCL2-OmPrAd, confirming stable transfection (Fig. 5a). In fact, gene expression of CXCL2 was significantly lower in the shCXCL2-OmPrAd than in the shNT-OmPrAd and non-treated control OmPrAd conditions (Fig. 5b).

Each type of OmPrAd with RFP was subcutaneously injected into NSG mice, and luciferase-expressing AGS was injected subcutaneously at its vicinity 1 week later. The tumours were finally excised from the mice at 11 weeks after injection. When GC tumours were excised, the adjacent fat tissues expressed RFP signals, suggesting that each OmPrAd could differentiate to human omental fat tissues (OmAd) in NSG mice (Fig. 5c). There were no significant differences in body weight between the two groups during the study period (Fig. 5d).

GC tumour growth with shCXCL2-OmAd was significantly lower than when co-injected with shNT-OmAd (Fig. 5e, f). In GC tumours with shNT-OmAd, the bioluminescence was detected in surrounding area around tumour, as well as tumour itself. In addition, the bioluminescence signal showed significantly lower intensity in GC tumours with shCXCL2-OmAd compared with those with shNT-OmAd (Fig. 5g, h). Gene expression of *VEGFA* was also decreased in GC tissues with shCXCL2-OmAd compared with GC tissues with shNT-OmAd (Fig. 5i). Immunoreactivity to Ki67 was significantly lower in GC tissues with shCXCL2-OmAd than in those with shNT-OmAd (Fig. 5j, k). Tumour angiogenesis was also significantly increased in GC tissues with shCXCL2-OmAd than in those with shNT-OmAd (Fig. 5l, m). Serum levels of human CXCL2 were lower in the shCXCL2-OmAd group compared with the shNT-OmAd group (Fig. 5n).

Lastly, we analysed circulating urinary levels of CXCL2 and VEGFA in GC patients with (*N* = 13) or without peritoneal metastasis (*N* = 75). Baseline characteristics were well balanced between two groups (Supplementary Table 2). Consistent with the in vitro and in vivo results, urinary levels of CXCL2 and VEGFA were significantly higher in GC patients with peritoneal metastasis than in those without peritoneal metastasis (Fig. 5o, p).

## Discussion

Using a multipronged approach, this study clearly demonstrated that omental adipocytes promote tumour growth and peritoneal dissemination of GC. Although peritoneal metastasis results in poor prognosis and various symptoms for advanced GC, the mechanism by which GC induces peritoneal metastasis remains unknown. This study sheds light on the role of omental adipocytes in peritoneal metastasis of GC.

There have been only two reports focusing on the omentum as a critical source for peritoneal metastasis of GC.^24,25^ The first study showed that intraperitoneally injected GC cells preferentially infiltrated into omental milky spots, comprising numerous macrophages and lymphocytes, in a mouse model.^24^ However, this study did not elucidate the mechanism in vitro and did not analyse whether and how the omentum facilitates peritoneal metastasis. The second study contained a similar concept to ours: that omental adipocytes play important roles in omental metastasis of GC.^25^ This study demonstrated that co-culture with omental adipocytes promotes cell invasion and facilitates accumulation of oleic acid into GC cells in vitro. The study concluded that oleic acid accumulation in GC cells might be critical, as GC cells stimulated by oleic acid induced GC cell invasion through PI3-AKT pathway activation.^25^ However, since this study has not directly demonstrated omental adipocyte-inducible peritoneal metastasis, the responsible mechanism remains unclear. In addition, no animal study was conducted. Hence, ours is the first study to report the significance of omental adipocytes for peritoneal metastasis of GC based on various mechanistic studies.

Importantly, the present orthotopic tumour model demonstrated that implanted GC cells significantly increased tumour growth and peritoneal dissemination in the omentum-preserved group compared with the omentectomy group. These results suggest that the omentum facilitates GC tumour growth and peritoneal dissemination. Complete omentectomy is generally recommended in (sub)total gastrectomy for resectable GC, although this is currently under debate.^26,27^ This conventional surgical approach may have contributed to a reduction in GC recurrence with peritoneal dissemination. Moreover, the present in vitro study revealed that omental adipocytes promoted cell proliferation, migration and capacity for angiogenesis in two non-metastatic GC cell lines. This malignant transformation of GC cells was regulated by the CXCL2–VEGFA axis through cell-cell communication between omental adipocytes and GC cells. In addition, the present animal study, including xenograft and humanised omental adipocyte models, supported this evidence.

CXCL2, which is also known as GROβ and macrophage inflammatory protein (MIP)-2α, is a member of the CXC chemokine family. CXCL2 is produced in response to infection or injury by various cell types including macrophages and is known to act as a chemotactic factor that attracts neutrophils by binding receptors, including CXCR1 and CXCR2.^28,29^ In addition, a previous study showed that CXCL2 induced angiogenesis through VEGFA release from neutrophils^30^ and promoted angiogenesis, cell migration and tumour growth in a hepatic metastasis of colon cancer model.^31^ The results of our GC study were also in line with these previous studies. In terms of adipocytes, it has been reported that CXCL2 secretion was highly elevated in differentiated adipocytes^32^ and obese human adipocytes.^33^ A recent study reported that born marrow adiposity induced bone-destruction bearing prostate cancer cells through increased secretion of CXCL1 and CXCL2.^34^ However, little is known about the potential roles of CXCL2 in adipocytes. Hence, our present study first demonstrated the critical roles of CXCL2 in omental adipocytes for tumour growth, angiogenesis and peritoneal dissemination of GC cells. CXCL2 represents a potential therapeutic target for peritoneal metastasis of GC. Moreover, this study has also demonstrated that urinary levels of CXCL2 was significantly higher in GC patients with peritoneal metastasis than in those without. Urinary level of CXCL2 may be a predictive biomarker for the presence of peritoneal metastasis.

Similar to GC, ovarian cancer is also characterised by extensive and rapid peritoneal metastasis. Some studies have described the mechanism of preferential peritoneal metastasis for ovarian cancer in the context of adipocyte microenvironment. The original study demonstrated that omental adipocytes promote homing and invasion of ovarian cancer cells and that interleukin-8 (IL-8) mediated these activities. Omental adipocytes also acted as an energy source for cancer cells.^18^ Interestingly, IL-8 shares the same receptors, CXCR1 and CXCR2, with CXCL2. The other study showed that omental adipocytes overexpressed salt-inducible kinase 2 in ovarian cancer cells, which induced cell proliferation and peritoneal metastasis thorough the PI3K-AKT pathway.^19^ The omental adipocyte-induced AKT phosphorylation was consistent with our results, which may be a key signal for omental adipocyte-induced cancer progression. Recent studies also demonstrated that omental adipocytes alter tumour metabolism in ovarian cancer cells, inducing tumour progression and peritoneal metastasis.^35,36^ These previous studies of ovarian cancer might support our results that omental adipocytes play crucial roles for induction of peritoneal metastasis in GC.

Although we focused on adipocytes and endothelial cells as the tumour microenvironment in this study, the human body possesses a more complex microenvironment, including immunogenic cells, mesenchymal stem cells and fibroblasts. However, our approach, which focused on omental adipocytes as the largest microenvironment for GC, could clearly present novel evidence of omental adipocyte-triggered peritoneal metastasis in GC.

**Fig. 6:**
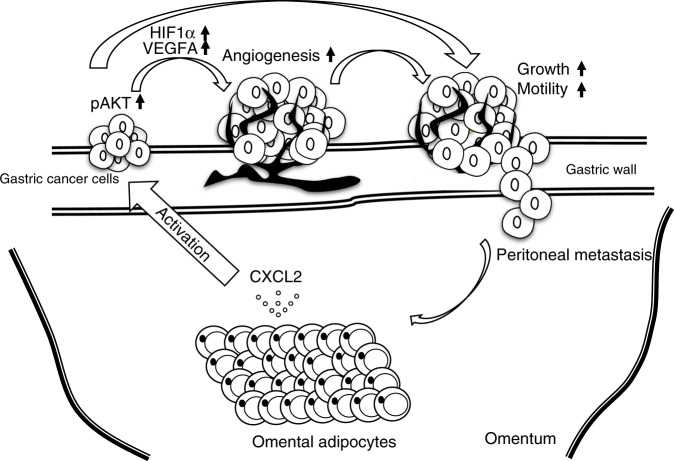
Proposed model of interaction between omental adipocytes and GC growth. CXCL2 secreted from omental adipocytes activates AKT phosphorylation of gastric cancer cells, which directly promotes gastric cancer growth and motility. In addition, VEGFA in gastric cancer cells is also upregulated through HIF1α upregulation, resulting in angiogenesis. Consequently, gastric cancer cells were transformed to a more aggressive phenotype, which induces peritoneal metastasis thorough recruitment to the omentum itself.

In conclusion, we highlighted omental adipocytes as a vast tumour microenvironment that connects to the stomach for peritoneal dissemination of GC in this study. Figure 6 summarises the schema of omental adipocyte-induced tumour growth and peritoneal metastasis. CXCL2 secreted from omental adipocytes stimulates primary GC tumours through AKT phosphorylation of GC cells, which directly promotes GC growth and invasion. In addition, GC cells after omental stimulation transform to an angiogenic phenotype through HIF1α and VEGFA overexpression, which also induces tumour growth and invasion. Eventually, GC shows peritoneal metastasis via these synergistic cross-talk effects between omental adipocytes and GC cells. CXCL2 might be a potential therapeutic target to regulate or prevent peritoneal dissemination of GC.

## Supplementary information

Supplementary File

## Data Availability

The data that support the findings of this study are available on request from the corresponding author (T.S.).
