# Supplementary material for: Omental adipocytes promote peritoneal metastasis of gastric cancer through the CXCL2–VEGFA axis
Source: Br J Cancer. 2020 May 22;123(3):459–70. doi: 10.1038/s41416-020-0898-3 (PMC7403422; doi:10.1038/s41416-020-0898-3)
Supplement: Supplementary file 1 — Supplementary File [file 41416_2020_898_MOESM1_ESM.pdf]

Supplementary File

MATERIALS and METHODS

Western blotting

Cultured GC cells were washed with PBS and resuspended in serum-free media. After overnight culture, the medium was replaced to individual CMs. After 24 h, cells were rinsed with PBS and disrupted in cell lysis buffer (Cell Signaling Technology, Danvers, MA) supplemented with *phenylmethylsulfonyl* fluoride (Sigma-Aldrich) for 5 min. Each sample was normalized against an equal protein concentration using a protein assay (BioRad, Hercules, CA) and then added to an equal quantity of sample buffer. Samples were heated to 100 °C for 5 min and mounted samples were separated by a 10% poly-acrylamide gel using a TGX™ FastCast™ Acrylamide kit (BioRad) and membrane-transferred. The membrane was blocked with 5% skim milk in phosphate buffered saline Tween 20 (PBST) for 1 h at room temperature, and the membrane was incubated with the following primary antibodies: Human (C-X-C motif chemokine receptor 1) CXCR1 antibody (R&D Systems) 1:500, human CXCR2 antibody (R&D Systems) 1:500, phospho-AKT (S473) antibody (Cell Signaling Technology) 1:1000, AKT antibody (Cell Signaling Technology) 1:1000 and β-actin monoclonal antibody (WAKO) 1:5000 overnight at 4 °C. The membranes were washed 3 times in PBST for 5 min each and then incubated with secondary antibody for anti-rabbit IgG, HRP-linked antibody1:5000 (Cell Signaling Technology) or anti-mouse IgG, HRP linked antibody 1:4000 (Cell Signaling Technology) for 1 h. Chemiluminescence was measured using an analyzer, GE ImageQuant LAS-4000mini (GE Healthcare, Chicago, IL).

Immunohistochemistry and immunofluorescence

Immunohistochemical staining was performed using an anti-Ki67 antibody (1:200) (Abcam, Cambridge, UK) and an anti-CD31 antibody (1:100) (Cell Signaling Technology, Inc. Danvers, MA), respectively. Consecutive 4-μm-thick sections were deparaffinized and hydrated through a graded alcohol series. After heating in 10 mM citrate buffer (PH6) in a microwave oven for 10 min at 98 °C, the samples were rinsed with PBS and then immersed in 3% hydrogen peroxide. Next, the sections were rinsed with PBS and blocked with 10% skim milk in PBST for 20 min at room temperature. Then, sections were incubated overnight with primary antibodies. After sections were rinsed with PBS, samples were incubated with biotinylated secondary antibody of EnVision+ System- HRP Labelled Polymer Anti-mouse (Agilent Technologies, Inc., Santa Clara, CA). After the sections were rinsed with PBS, the samples were incubated in 0.01% H_2O_2 and 0.05% 3,3'-diaminobenzidine tetrachloride. Nuclear counterstaining was accomplished with hematoxylin.

RFP expression in sections of GC tumors injected in the vicinity of shNT-OmAd or shCXCL2-OmAd was detected by fluorescence microscopy. The tumors were frozen and fixed with xylene.

Supplementary Table 1. Primers used in this study

Gene	Forward primer	Reverse primer
CXCL1	CATCGAAAAGATGCTGAACAGT	CTTCAGGAACAGCCACCAGT
CXCL2	TCTCAACCCCGCATCGC	ACAGCCACCAATAAGCTTCC
CXCL3	AGATACTGAACAAGGGGAGCAC	CACCCTGCAGGAAGTGTCAA
β-actin	ACAGAGCCTCGCCTTTGC	GCGCGGCGATATCATCATCC
VEGFA	GCAGAAGGAGGAGGGCAGAAT	GCACACAGGATGGCTTGAAGA
VEGFD	CTGCCTGATGTCAACTGCTTAG	TGAGATGATCGCTTCACTGGTC
ANGPTL4	CCACTTGGGACCAGGATCAC	CGGAAGTACTGGCCGTTGAG
INFB1	ACGCCGCATTGACCATCTAT	GTCTCATTCCAGCCAGTGCT

CXCL1, C-X-C motif chemokine ligand 1; *CXCL2*, C-X-C motif chemokine ligand 2; *CXCL3*, C-X-C motif chemokine ligand3; *VEGFA*, vascular endothelial growth factor A; *VEGFD*, vascular endothelial growth factor D; *ANGPTL4,* angiopoietin-like 4; *INFB1*, interferon beta;

Supplementary Table 2. Patients' characteristics for urinary analysis

		**Non-metastasis (n=75)**	**Peritoneal metastasis (n=13)**	***P***
Age (years)	Median (range)	68 (44-85)	73 (47-86)	0.133
Sex	Male	51	9	1.000
	Female	24	4	
Serum Cr (mg/dl)	Mean±SD	0.77±0.18	0.74±0.20	0.795
Location	Cardia	4	1	0.559
	Body	71	12	

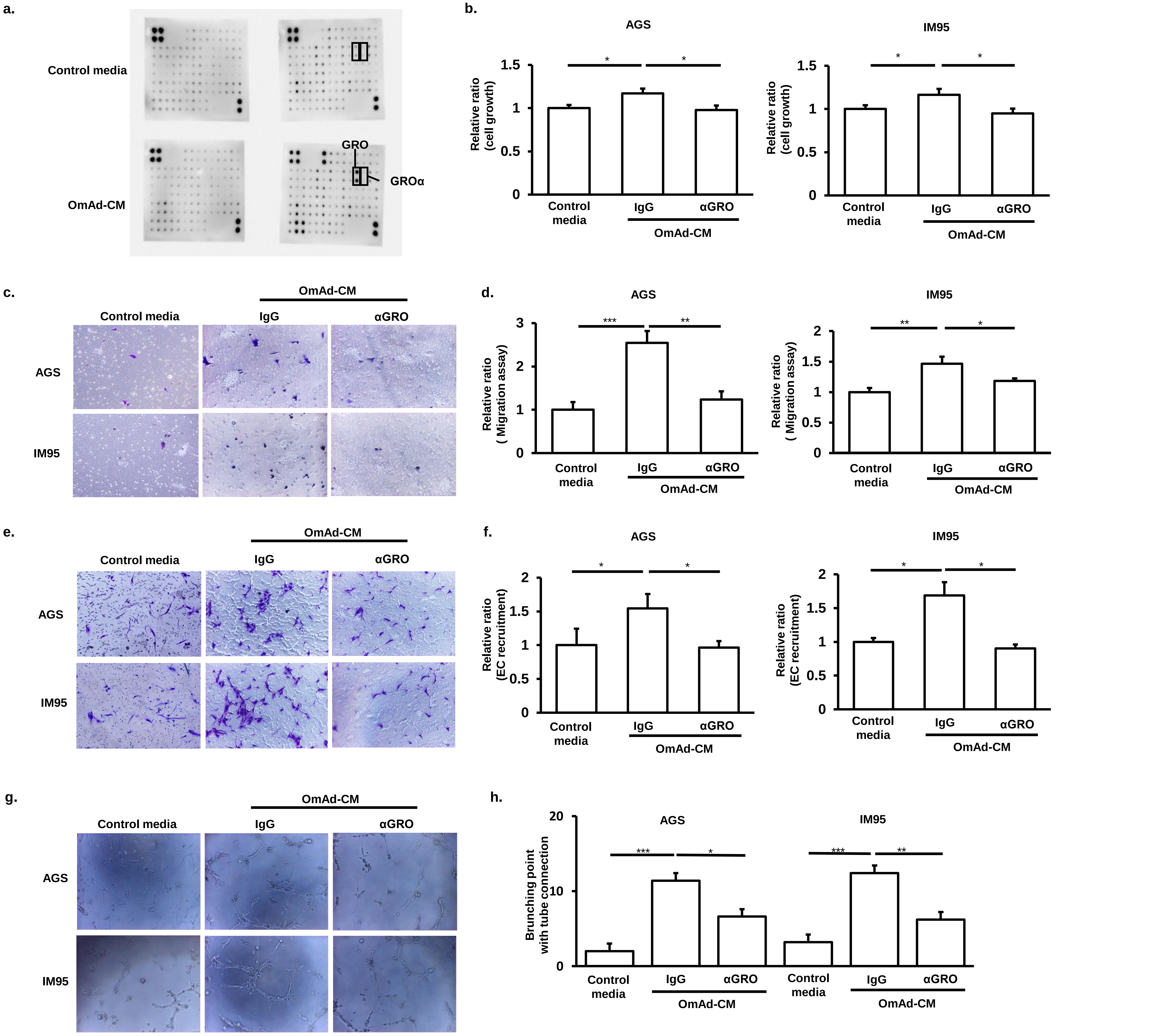

Supplementary Fig. 1 GRO antibody attenuates omental adipocytes-induced GC cell growth/migration and *in vitro* angiogenesis.
Each graph represents the mean ± SE from three independent experiments. *, P<0.05; **, P<0.01; ***, P<0.001

a. Cytokine antibody array. upper membranes: control media, lower membranes: OmAd-CM

b. Cell growth. AGS and IM95 cells were incubated with control media or OmAd-CM treated with IgG (10 µg/ml) or GRO antibody (10 µg/ml) (n = 5). Mean, AGS: 1.0 (control), 1.2 (IgG), 1.0 (αGRO); IM95: 1.0 (control), 1.2 (IgG), 1.0 (αGRO).

c. Representative images of migration assay (×100). d. Quantification of migration assay. Migrated GC cells were counted from averages at four microscopic fields, and each result was presented as the mean of at least three independent experiments. Each value represents the mean relative ratio of migrated GC cells under OmAd-CM treated with IgG (10 µg/ml) or GRO antibody (10 µg/ml) to those under control media. Mean, AGS: 1.0 (control), 2.5 (IgG), 1.2 (αGRO); IM95: 1.0 (control), 1.5 (IgG), 1.2 (αGRO).

e. Representative images of EC recruitment assay (×100). f. Quantification of EC recruitment assay Migrated HMVECs were counted from averages at four microscopic fields, and each result was presented as the mean of at least three independent experiments. Each value represents the mean relative ratio of migrated HMVECs co-cultured with GC cells treated with OmAd-CM including 10 µg/ml IgG or 10 µg/ml anti-GRO antibody to those co-cultured with GC cells treated with control media. Mean, AGS: 1.0 (control), 1.5 (IgG), 1.0 (αGRO); IM95: 1.0 (control), 1.7 (IgG), 0.9 (αGRO).

g. Representative images of tube formation assay (×100). h. Quantification of tube formation assay. Each value represents the mean number of branched tubes under each condition. Mean, AGS: 2.0 (control), 11.4 (IgG), 6.6 (αGRO); IM95: 3.4 (control), 12.4 (IgG), 6.2 (αGRO). IgG (10 µg/ml); GRO antibody (10 µg/ml) .
